# Factors associated with good neurological outcomes in survivors of in-hospital cardiac arrest: A systematic review and meta-analysis

**DOI:** 10.1016/j.ccrj.2026.100202

**Published:** 2026-07-28

**Authors:** Ramon Joel Seastres, Ogee Mer Panlaqui, Shaun Khanna, Alice Leung, Ramon Jose Seastres, Mohammad Mobashir, Victoria Ying, Aditya Bhat, Dhaval Ghelani, Graham Philip Reece, Fabio Silvio Taccone

**Affiliations:** a Department of Intensive Care Unit, Blacktown Hospital, Sydney, NSW, Australia; b Department of Cardiology, Blacktown Hospital, Sydney, NSW, Australia; c Monash Newborn, Monash Children's Hospital, Victoria, Australia; d Department of Intensive Care Unit, Hôpital Universitaire de Bruxelles (HUB), Université, Libre de Bruxelles (ULB) Brussels, Belgium

**Keywords:** In-hospital cardiac arrest, Systematic review, Meta-analysis, Neurological outcome, In-Hospital Cardiac Arrest, Heart arrest, Neurological Outcome, Glasgow outcome scale, Survivor, Humans

## Abstract

**Background:**

Accurate predictors of neurological outcome after in-hospital cardiac arrest (IHCA) remain limited.

**Methods:**

We conducted a systematic review and meta-analysis of 15 studies (n = 4948 adults) to identify peri-arrest factors associated with favourable neurological outcomes in IHCA survivors. Study quality was assessed using the QUIPS tool and evidence certainty was rated with GRADE.

**Results:**

A favourable neurological outcome was associated with pre-arrest factors such as male sex (odds ratio [OR]: 1.42, 95% confidence interval [CI]: 1.13–1.78, Level of Evidence (LOE): low), established ischaemic heart disease (OR: 1.84, 95% CI: 1.12–3.02 LOE: low), acute myocardial infarction diagnosis upon hospital admission (OR: 2.14 95% CI: 1.53–2.99 LOE: low), acute heart failure (OR: 1.99, 95% CI: 1.42–2.78 LOE: low) and arrests in telemetry units (OR: 1.34, 95% CI: 1–1.78 LOE: low). Initial shockable rhythm (OR: 3.0, 95% CI: 2.3–3.9, LOE: moderate), cardiac-related causes (OR: 2.12, 95% CI: 1.54–2.92, LOE: low to moderate) and shorter cardiopulmonary resuscitationduration (standardised mean difference: -0.62, 95% CI: -0.89, −0.35) were also associated with improved neurological recovery. Post-arrest interventions such as emergent coronary catheterisation (OR: 4.55, 95% CI: 2.11–9.82, LOE: low), pulmonary artery catheterisation monitoring (OR: 3.84, 95% CI: 1.51–9.75, LOE: low), and extracorporeal membrane oxygenation after return of spontaneous circulation (OR: 1.71, 95% CI: 1.20–2.43, LOE: low) were associated with better neurological outcomes. The overall quality of included studies was generally of low certainty, limited by moderate bias and substantial heterogeneity.

**Conclusions:**

Early detection and management of reversible cardiac causes in IHCA are associated with improved patient outcomes. This study identifies peri-arrest factors associated with favourable neurological outcomes in adult survivors of IHCA who were discharged alive. All findings and interpretations are limited to this conditional population and should not be extrapolated to the broader IHCA cohort. While these findings offer valuable insights for risk stratification and protocol development, high-quality prospective studies are needed to validate these associations and confirm their clinical significance.

## 1 Introduction

Despite advancements in resuscitation techniques, improving survival rates for in-hospital cardiac arrest (IHCA) remains challenging. Clinical understanding of IHCA often relies on large data available on out-of-hospital cardiac arrest (OHCA). IHCA and OHCA represent distinct clinical entities with significant differences in terms of arrest characteristics, location, causes, response time, and survival outcomes. Recognising these differences is critical for directing appropriate care and improving prognosis**.** Recent registry data indicate that survival rates following IHCA remain modest, with one-year survival estimates ranging from 10% to 25% and favourable neurological outcomes reported in only 7%–15% of cases.^2^^,^^7^ These figures underscore the ongoing challenge of improving both survival and neurological recovery in the IHCA population. Early literature focused on survival rates while modern resuscitation research has shifted focus towards long-term functional neurological status as a primary metric for evaluating quality of life post-arrest. A rapid bystander CPR and defibrillation in OHCA predict good neurological outcome, while IHCA is better with underlying cardiac aetiology, younger age, shorter resuscitation times, lower serum lactate, and shockable rhythms.^1^

Cardiac-related arrests, from myocardial ischaemia, reported a higher 30-day survival and better outcome.^2^ However, long-term recovery is often hindered by post-cardiac arrest acute brain injury from prolonged lack of oxygen and reperfusion damage.^3^ The absolute rates of favourable neurological recovery remain an area of continued research.

This systematic review and meta-analysis aim to determine the peri-arrest factors (pre-arrest, intra-arrest, and post-arrest) associated with favourable neurological outcomes in adult IHCA patients who survived to hospital discharge.

## 2 Methods

Data collection and reporting followed the meta-analysis of observational studies in epidemiology reporting guideline as suggested by enhancing the quality and transparency of health research network. This meta-analysis study is exempt from ethics approval since the summarised and synthesised collected data were reported from previous clinical studies and from which informed consent had been obtained in prospective studies by the clinical investigators or waived due to the retrospective nature. This study was registered in the National Institute for Health–Research International Prospective Register of Systematic Reviews (PROSPERO ID CRD42024520780). A systematic literature search of medical databases (Medline, Embase, and Google Scholar) and grey literature using Preferred Reporting Items for Systematic reviews and Metaanalyses (PRISMA) principles was conducted from 2000 to 2022. The search strategy was developed with assistance of the university librarian from Peter Zelas Library Blacktown Mount Druitt Hospital NSW Australia by using keywords “cardiac arrest”, “cardiopulmonary resuscitation” and “hospitalization” or “in-hospital”, “neurological outcome”, and “prognostic factors”. IHCA is defined as cardiac arrest in persons who occupied a hospital bed such as in-patient or hospital admission, or intensive care unit. The reference lists of identified articles were manually searched for any additional relevant studies, whereas eligible studies were screened for completeness.

### 2.1 Eligibility criteria, outcomes, selection process and data collection process

We included all English language articles detailing retrospective and prospective observational studies, randomised controlled trials, and quasi-randomised controlled trials. The study population is restricted to adults (≥18 years) who achieved return of spontaneous circulation (ROSC) and survived to hospital discharge after IHCA. The study measures survival to hospital discharge with favourable or good neurological outcome. Favourable neurological outcome is defined by standardised scales: Glasgow–Pittsburgh cerebral performance category (CPC) 1-2, modified Rankin scale (mRS)≤3, Glasgow outcome scale (GOS) 4-5 at discharge or follow-up. A poor or unfavourable neurological outcome is defined as Glasgow–Pittsburgh Cerebral Performance Category (CPC) scale of 3–5 or mRS of 4–6 or GOS of 1–3. Data extraction was limited only to studies in IHCA patients. Studies were included if they reported any measures of neurological outcomes upon discharge or during subsequent follow-up. All interpretations, effect sizes, and associations are relevant only to the survivor cohort.

### 2.2 Study screening

The studies were screened independently by co-investigators at the title and abstract level using Covidence Systematic Review Software (Veritas Health Innovation Ltd, Melbourne, Australia) and titles were imported into Covidence directly from the search databases and duplicates were removed. The same reviewers also performed the full-text review while data extraction was obtained by another pair of investigators. Any aforementioned disagreements were discussed with the third investigator until a consensus was reached. The final selected studies were discussed with the senior research advisers prior to data extraction. Selected studies were evaluated for inclusion in the meta-analysis while duplicated data, incomplete data, and other clear selection bias were excluded. The following information was collected from each study such as study characteristics (author, year published, and study design). The study population and its relevant factors were defined and described in all studies. The means of outcome measurement is satisfactorily described. The eligible studies were analysed and assessed by two independent investigators using QUIPS tool for evaluation of the quality of prognosis studies.

### 2.3 Statistical analysis

Statistical analyses were conducted using STATA MP-Parallel Edition Statistical Software, Version 18, College Station, TX: Stata Corp LP. A *p*-value ≤0.05 was considered statistically significant. The observational design of the selected studies was considered and the methodological differences were potentially responsible for a significant component of the variance within the measures of interest. The study did not assume one effect size among the included studies, thus the overall effect for each meta-analysis was estimated using a random-effects model (REM), which considers within-study and between-study variations (Sterne, 2009). Nevertheless, a fixed-effect model (FEM) was employed if the estimated heterogeneity was not substantial (<50%). Articles were required to report on a quantitative estimation including mean, confidential interval (CI), and standard deviation. 95% confidence intervals (CIs) were calculated for all indices. Pooled odds ratio (OR) derived from adjusted estimates were obtained across studies. The peri-arrest factors affecting neurological outcomes were represented using OR alongside their corresponding 95% CIs. CPR duration will be analysed using both standardised mean difference (SMD) and OR effect measures. SMD will be used for studies reporting mean CPR duration by outcome group, while OR will derive from studies reporting CPR duration as a continuous predictor. These metrics will be reported separately and are not interchangeable. The log OR and associated standard errors of individual studies reporting continuous-level predictors (age and duration of CPR) will initially be estimated and were then analyse using the meta set command of STATA. The pooled log ORs, and their 95% CIs, will be converted to OR to yield the per-minute OR for interpretability. Heterogeneity was appraised using the following statistical tests: Q-statistics test, *I*^*2*^ statistics, and tau-squared (*τ*^*2*^) statistics (Higgins, 2003). *I*^*2*^ values greater than 50% imply substantial heterogeneity (Higgins, 2003), while Q-statistics with a significant *p*-value denotes a statistically significant heterogeneity. High I^*2*^ values (>75%) limit confidence in the precision of the pooled estimate due to significant inconsistency in the effect size. Publication bias was graphically analysed using contour-enhanced funnel plots. Formal statistical assessments of funnel plot asymmetry were performed using Egger’s regression asymmetry test and Begg’s adjusted rank correlation test.

## 3 Results

### 3.1 Study selection and characteristics of the included study

A detailed flow chart of the study selection process is described (Supplementary Fig. S1–S5). A total of 428 studies were screened and 15 peer-reviewed articles^2^^,^^4^^,^^6^^,^^9^^,^[14], [15], [16], [17], [18], [19], [20], [21], [22], [23], [24] were analysed encompassing 4948 adults and 39 identified clinical factors. There were a total of 15 observational studies (4 prospective and 11 retrospective) conducted across Asia, Australia, North America, and Europe (Supplementary Table S1, Supplementary Table S2, Supplementary Table S3). The mean patient age was 63 years (58-71, standard deviation [SD]: 3.49), mostly male gender (65%). The overall quality of included trials demonstrated a moderate risk of bias and low to moderate level of certainty (Supplementary Tables S5–S10).

### 3.2 Pre-arrest factors and neurological outcome

Favourable neurological outcomes after IHCA were associated with several pre-arrest characteristics such as male sex (OR: 1.45, 95% CI: 1.14–1.79 *p* = 0.002) (Table 1), cardiac-related causes (OR: 2.12, 95% CI: 1.54–2.92, *p* = 0.001, Fig. 1a) and arrest in telemetry units (OR: 1.34, 95% CI: 1.00–1.78, *p* = 0.048, Fig. 1c). Specific cardiac conditions, including chronic ischaemic heart disease (OR: 1.84, 95% CI: 1.12–3.02, *p* = 0.016), acute heart failure (OR: 1.99, 95% CI: 1.42–2.78, v = 0.001) (Table 1), and acute myocardial infarction during admission (OR: 2.14, 95% CI: 1.53–2.99, v = 0.001, Fig. 1b) were associated with favourable neurological recovery. Lower probability of favourable neurological outcome were observed in malignancy (OR: 0.23, 95% CI: 0.10–0.52, *p* = 0.001), liver disease (OR: 0.62, 95% CI: 0.44–0.88, *p* = 0.007), respiratory insufficiency (OR: 0.40, 95% CI: 0.21–0.75, *p* = 0.004), Sepsis (OR: 0.49, 95% CI: 0.32–0.74, *p* = 0.001) and high Charlson Comorbidity Index (SMD -0.57, 95% CI -0.75,0.39, *p* = 0.001) (Table 2). All other variables were not significantly associated with neurological outcomes (Table S4 Supplementary).

**Table 1:** Factors of IHCA associated with good neurologic outcome.

Pre-arrest factors							

	Studies	Patients (n)	Pooled OR	95% CI	*p*-value	I2 (%)	Grade certainty

Male gender	15	4948	1.425	1.136,1.788	0.002	46%	Low
Cardiac cause	6	1733	2.12	1.54,2.92	0.001	46%	Low to moderate
Chronic ischaemic heart disease	6	2627	1.84	1.12, 3.02	0.016	38%	Low
Acute heart failure	6	1981	1.99	1.42,2.78	0.001	0	Low
Acute myocardial infarction	5	2469	2.14	1.53,2.99	0.001	0	Low
Arrest in telemetry	10	3629	1.34	1.00,1.78	0.048	44%	Low

**Intra-arrest factors**							

	**Studies**	**Patients (n)**	**Pooled OR**	**95% CI**	*p***-***v***alue**	**I2 (%)**	

Initial rhythm VF/VT	13	4282	3.0	2.30, 3.91	0.001	48%	Moderate

**Post-arrest factors**							

	**Studies**	**Patients (n)**	**Pooled OR**	**95% CI**	*p***-value**	**I2 (%)**	

Use of PA catheter	4	1957	3.84	1.51, 9.75	0.005	0	Low
Emergent coronary catheterisation	7	2713	4.55	2.11, 9.82	0.001	80%	Low
ECMO after ROSC	7	2985	1.71	1.20, 2.43	0.003	0	Low

CI: confidence interval; ECMO: Extracorporeal membrane oxygenation; IHCA: in-hospital cardiac arrest; OR: odds ratio; PA: pulmonary artery; ROSC: return of spontaneous circulation; VF: ventricular fibrillation; VT: ventricular tachycardia.

**Fig. 1(a):**
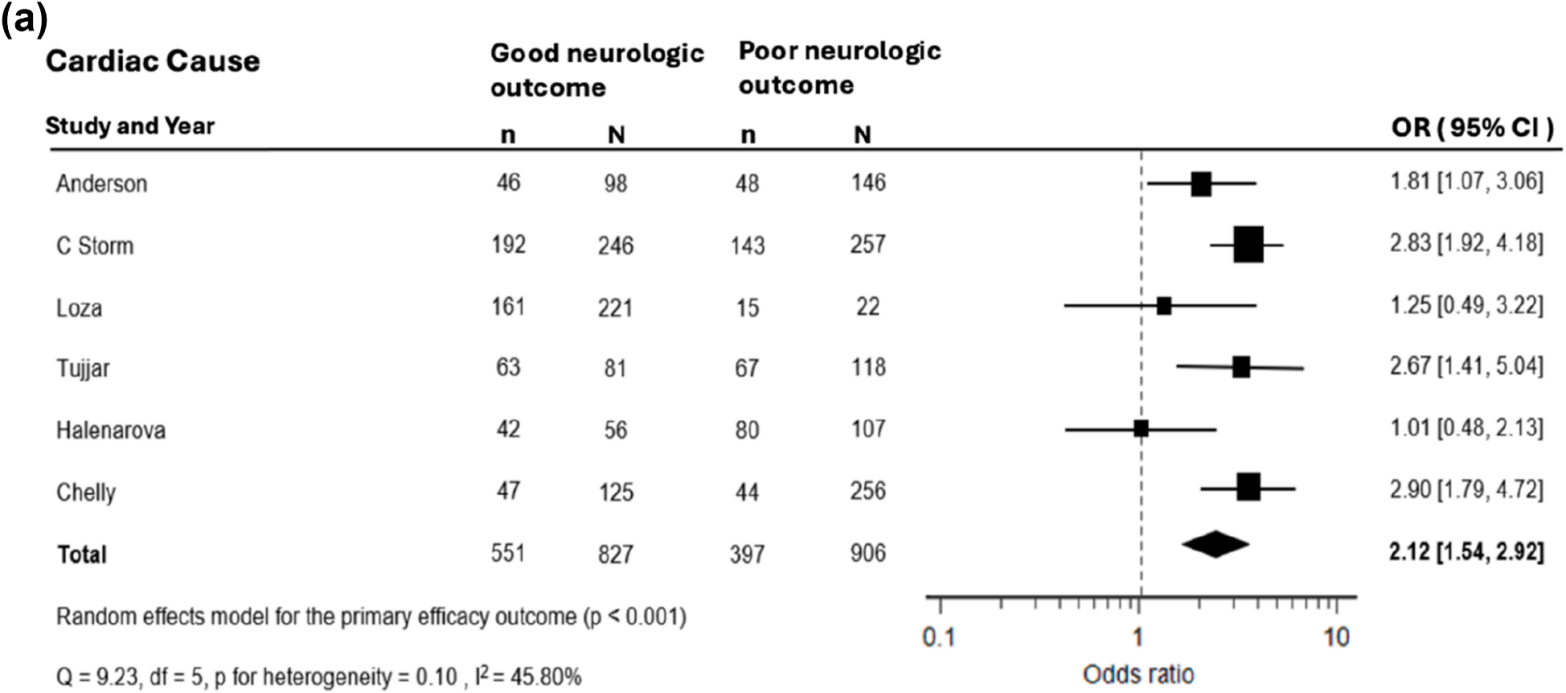
Cardiac cause.

**Fig. 1(b):**
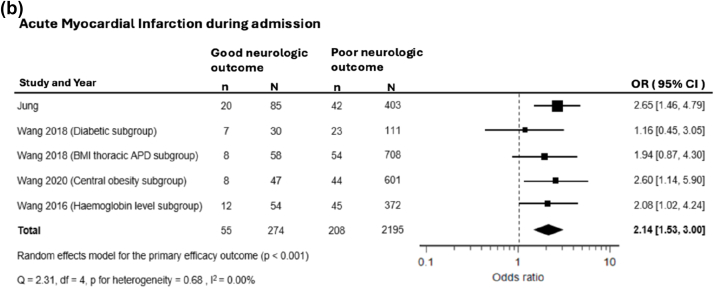
Acute myocardial infarction during hospital admission.

**Fig. 1(c):**
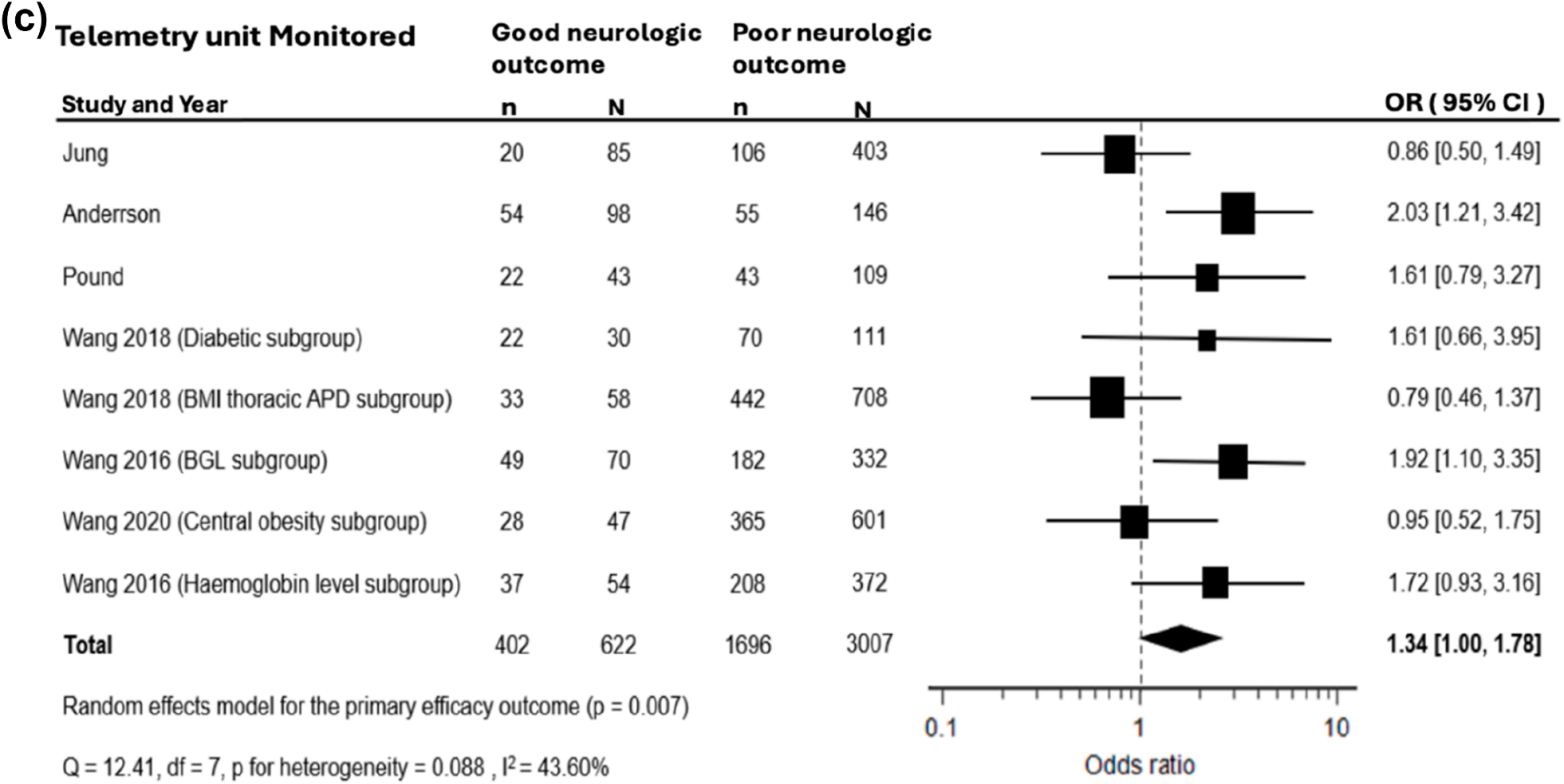
Arrest in telemetry.

**Table 2:** Factors of IHCA associated with a poor neurologic outcome.

Prearrest factors							

	Studies	Patients (n)	Pooled OR	95% CI	*p*-value	I2 (%)	Level of certainty

Malignancy	7	3115	0.23	0.10, 0.52	0.001	73%	Low
Liver disease	8	3233	0.62	0.44, 0.88	0.007	12%	Low
Respiratory insufficiency	8	3233	0.40	0.21, 0.75	0.004	84%	Low
Sepsis	6	2871	0.49	0.32, 0.74	0.001	0	Low

	**Studies**	**Patients (n)**	**Pooled SMD**	**95% CI**	*p*-v**alue**	**I2 (%)**	**Level of certainty**

Old age	14	3078	−0.18	−0.28, −0.08	0.001	0	Low
High Charlson Comorbidity index	3	1555	−0.57	−0.75, −0.39	0.001	0	Low to moderate
Low haemoglobin	2	1026	0.37	0.03, 0.71	0.03	39%	Low

**Intra-arrest factors**							

	**Studies**	**Patients (n)**	**Pooled OR**	**95% CI**	*p*-v**alue**	**I2 (%)**	**Level of certainty**

PEA arrest	6	1457	0.45	0.25, 0.81	0.008	78%	Low
Asystole arrest	4	971	0.63	0.42, 0.96	0.033	24%	Low to moderate

	**Studies**	**Patients (n)**	**Pooled SMD**	**95% CI**	*p*-v**alue**	**I2 (%)**	**Level of certainty**

Duration of CPR	14	2062	−0.55^a^	−0.69, −0.41	0.001	1%	Low to moderate

CI: confidence interval; CPR: cardiopulmonary resuscitation; IHCA: in-hospital cardiac arrest; OR: odds ratio; PEA: pulseless electrical activity; SMD: standardised mean difference.

ORs reflect per minute effects from studies reporting CPR duration as a continuous variable. These metrics are reported separately and are not interchangeable.

a SMD values reflect group level differences in mean CPR duration. Fixed-effect synthesis shown for comparability; primary random estimate were reported in Fig. 3b. Metrics are not interchangeable across models; Heterogeneity differs by design.

### 3.3 Intra-arrest factors and neurological outcome

Initial shockable rhythms such as ventricular fibrillation and ventricular tachycardia are associated with favourable neurological outcomes (OR: 3, 95% CI: 2.30–3.91, *p* < 0.001, Fig. 2a). Non-shockable rhythms, including pulseless electrical activity; OR: 0.45, 95% CI: 0.25–0.81, *p* = 0.008) and asystole (OR: 0.63, 95% CI: 0.42–0.96, *p* = 0.03, Table 2) were correlated with poorer outcomes. A shorter CPR duration associated with favourable neurological outcomes had a mean CPR duration of 10.99 min (SD: 8.45) compared to 19.4 min (SD: 16.96) with poorer outcome (SMD: -0.62, 95% CI: -0.89, −0.35; *p* = 0.001, I2 = 92%). The pooled OR for CPR duration was 0.80 (95% CI: 0.67–0.97) indicating that each additional minute of CPR was associated with a 20% reduction in the odds of a favourable neurological outcome among survivors. These effect measures are not interchangeable and were reported separately (Fig. 2(b), Fig. 2(c)b and c). The heterogeneity across studies was very high which limits the precision and reliability of the pooled estimates. We find it difficult to establish an accurate cut off point between favourable and unfavourable neurologic outcomes .

**Fig. 2(a):**
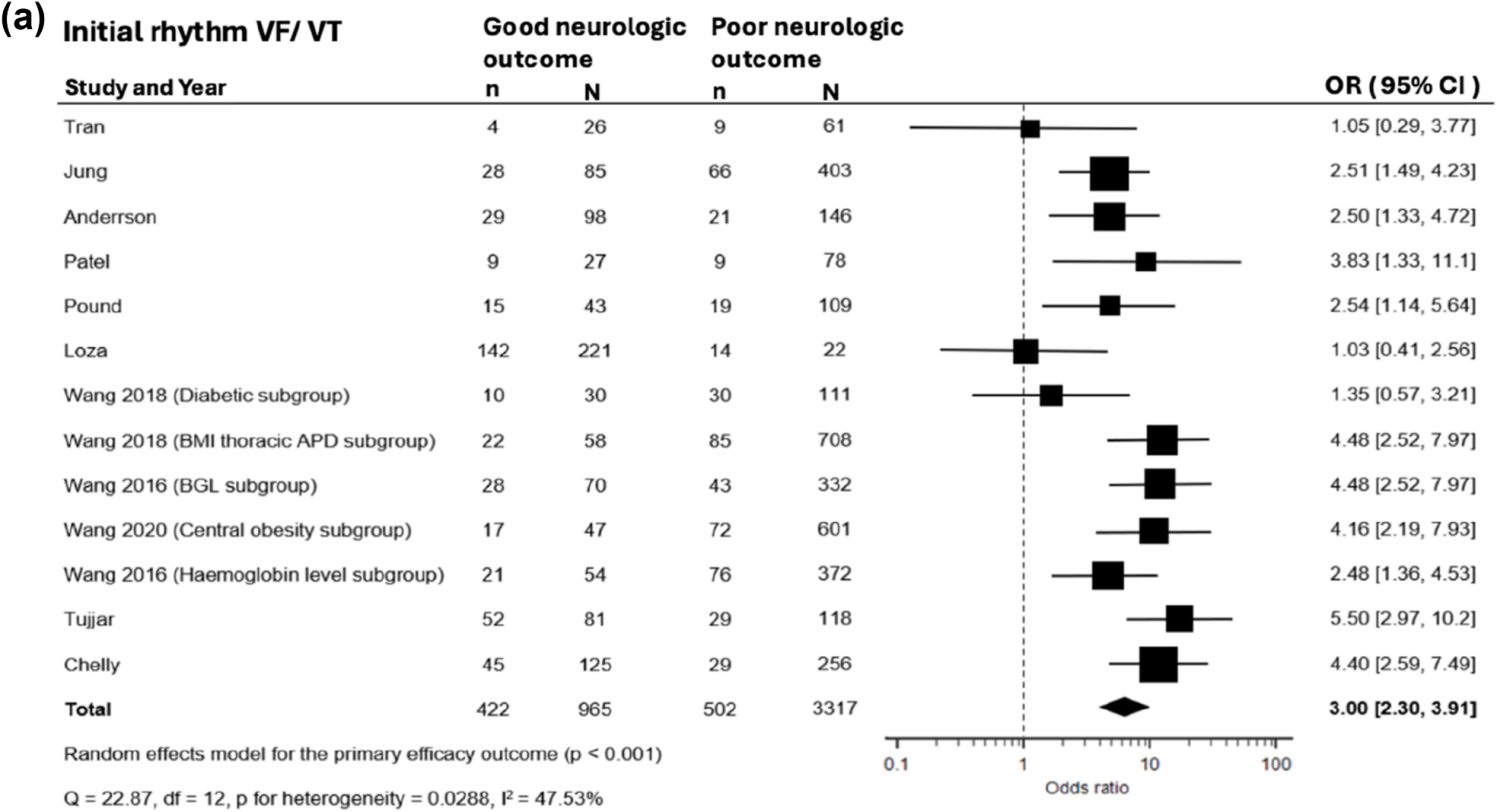
Initial shockable rhythm (VF/VT). VF: ventricular fibrillation; VT: ventricular tachycardia.

**Fig. 2(b):**
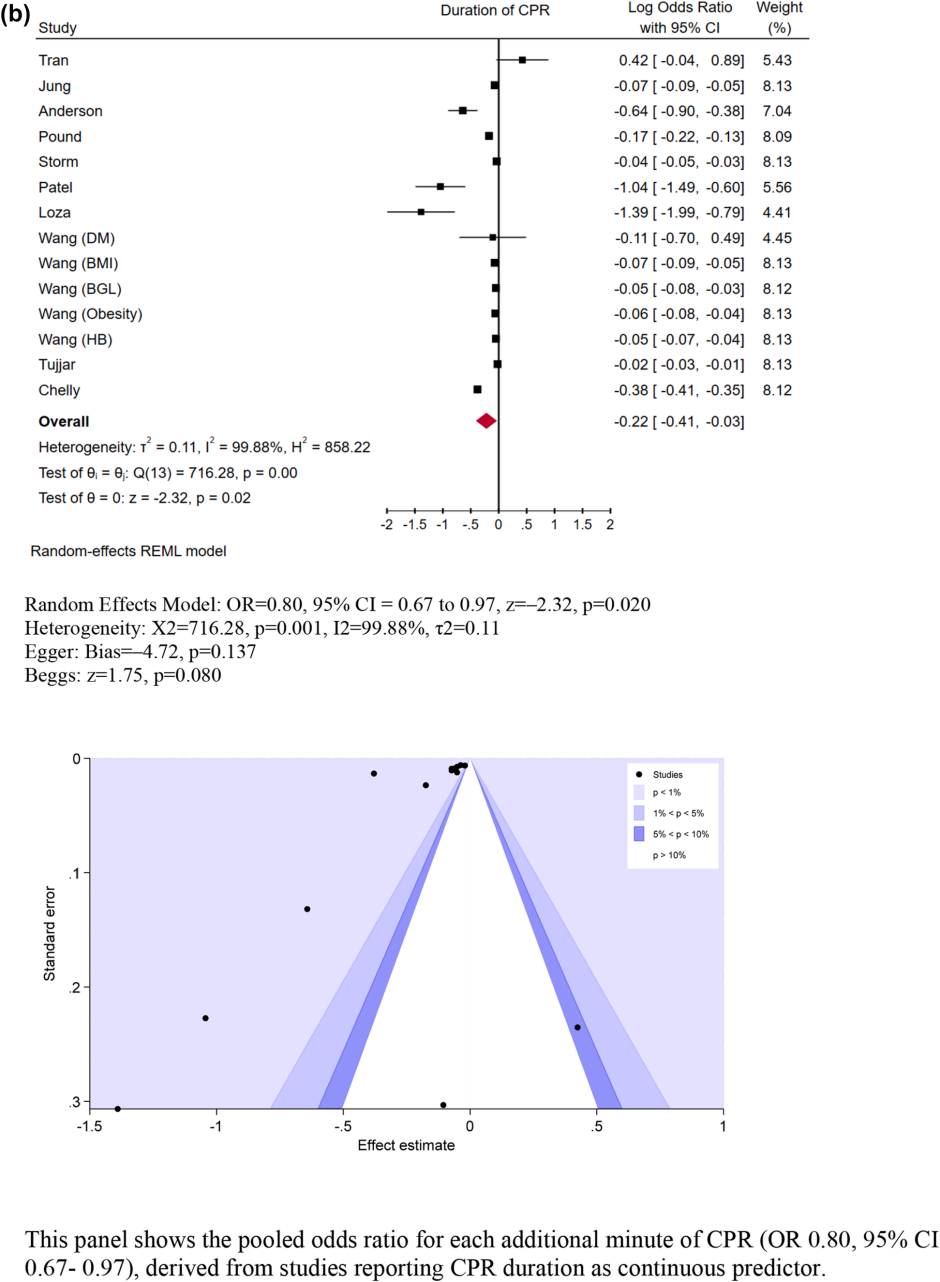
Duration of CPR: odds ratio per additional minute of CPR. CPR: cardiopulmonary resuscitation

**Fig. 2(c):**
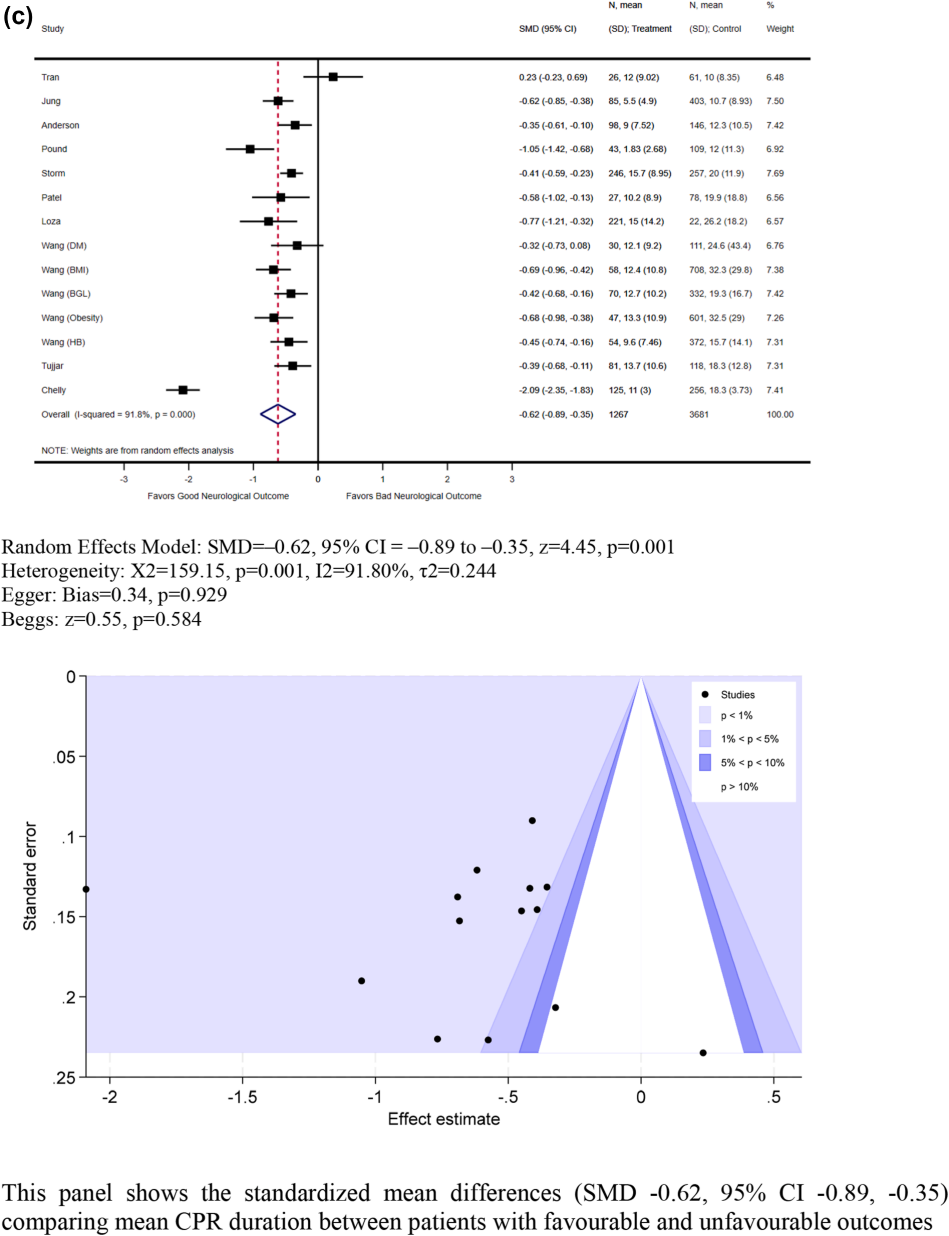
Duration of CPR: standardised mean difference in CPR duration between outcome groups. CPR: cardiopulmonary resuscitation.

**Fig. 3:**
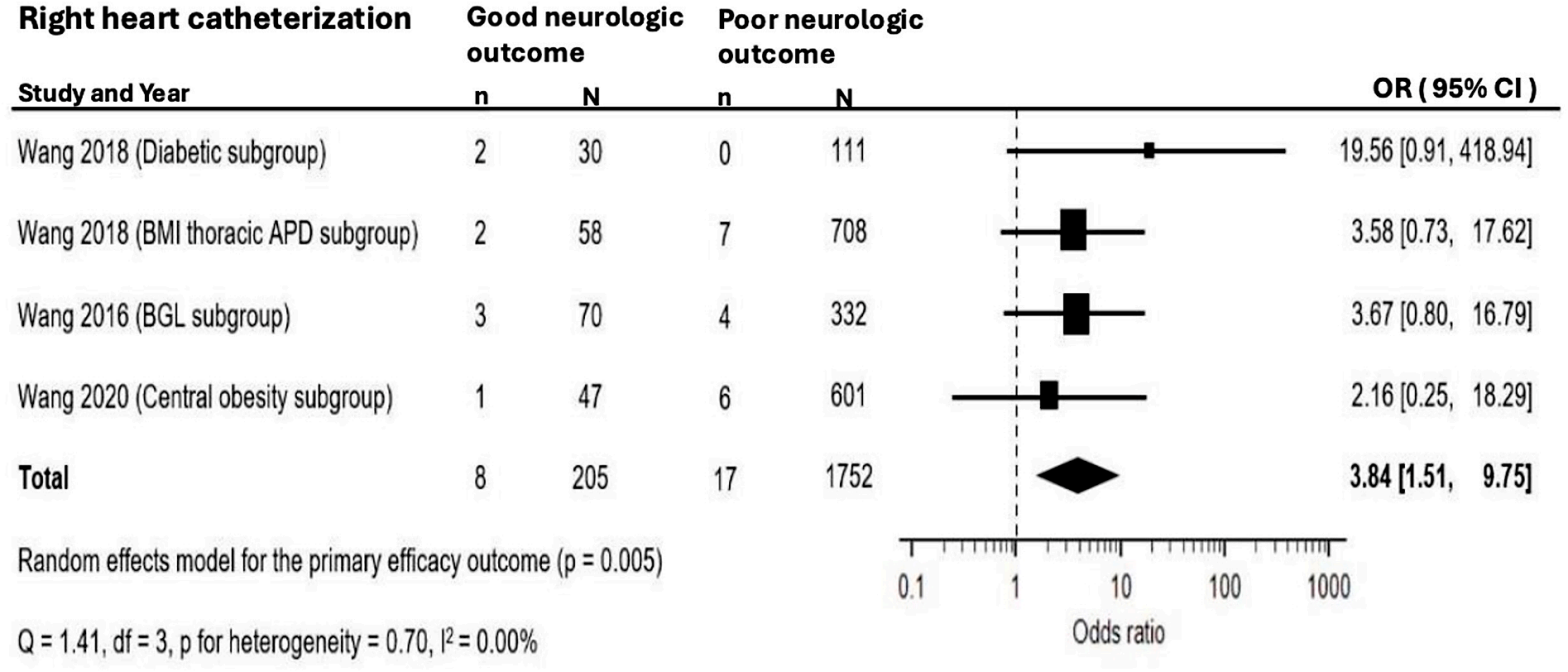
(a). **Initial shockable rhythm (VF/ VT).** (b). Duration of CPR: Odds ratio per additional minute of CPR. (c). Duration of CPR: Standardized mean difference in CPR duration between outcome groups.

### 3.4 Post-arrest factors and neurological outcome

Advanced post-arrest interventions like the use of pulmonary artery catheter monitoring (OR: 3.84, 95% CI: 1.51–9.75, *p* = 0.005), emergent coronary catheterisation (OR: 4.55, 95% CI: 2.11–9.82, *p* < 0.001) and extracorporeal membrane oxygenation (ECMO) after ROSC (OR: 1.71, 95% CI: 1.20–2.43, *p* = 0.003) were associated with better neurological outcomes (Table 1).

## 4 Discussion

This systematic review provides an in-depth analysis of factors (pre-arrest, intra-arrest, and post-arrest) associated with neurological outcomes among IHCA survivors. Favourable neurological outcomes in adult survivors of IHCA are associated with early recognition and management of reversible cardiac causes, initial shockable rhythm, arrest in telemetry, shorter CPR duration, and advanced post-arrest interventions such as ECMO, use of pulmonary artery catheter monitoring, and emergent coronary catheterisation, though evidence certainty is low and findings apply only to those discharged alive. Consistent with prior registry data, our findings indicate that rapid, targeted interventions for shockable rhythms and cardiac causes are associated with improved IHCA outcomes. However, this study extends these findings by evaluating post-arrest neurological function, offering a more precise understanding of favourable survival rather than simple survival rates.

### 4.1 Pre-arrest factors

Pre-arrest factors such as male gender, telemetry monitoring, and underlying cardiac conditions (ischaemic heart disease, acute heart failure, and myocardial infarction) are associated with better neurological recovery at discharge, though evidence is of low certainty. Male sex correlates with better outcome in survivors than in females. This finding is likely confounded by methodological limitations. Most of the included studies were observational and survivor-only cohorts, this improved neurological outcome may stem from selection bias rather than inherent biological differences. Advanced age and comorbidities such as malignancy, liver disease, respiratory failure, sepsis, and higher Charlson comorbidity scores are associated with poorer outcomes. IHCA patients in monitored hospital settings had better outcomes due to early detection, rapid response activation, and continuous haemodynamic monitoring. Conversely, non-monitored settings with non-cardiac arrests often experience delayed resuscitation and worse outcomes.[15], [16], [17]

### 4.2 Intra-arrest factors

Intra-arrest factors such as initial shockable rhythms, i.e. ventricular fibrillation or ventricular tachycardia, and shorter resuscitation times are associated with improved neurological recovery. Shockable rhythms, often stemming from primary cardiac causes, have higher survival rates due to the efficacy of early defibrillation. Updated guidelines emphasise rapid identification and management of reversible cardiac causes, early defibrillation for shockable rhythms, and minimisation of CPR duration as central components of IHCA protocols.^28^ The CPR efficiency diminishes rapidly over time and with prolonged CPR beyond 10 min, the odds of favourable neurological recovery declines every additional minute. While shorter CPR duration was associated with improved neurologic outcome, this should be interpreted with caution as it implies as a surrogate marker of resuscitation process such as no-flow time, low-flow time, CPR quality, and reversibility of the underlying cause.

### 4.3 Post-arrest factors

Our findings indicate that the application of advanced invasive post-resuscitation interventions is associated with better outcomes. By synthesising data across diverse clinical settings and patient populations, this study offers novel insights into the factors of neurological recovery. The study emphasised the need for targeted resuscitation and post-arrest care protocols tailored to specific patient profiles. Current guidelines suggest initial mean arterial pressure targets of at least 60–65 mm Hg with further adjustments based on individual patient status. Emergency coronary angiography is recommended for patients with ROSC and suspected cardiac aetiology presenting with ST elevation myocardial infarction, shock, or instability. Conversely, routine immediate angiography for all comatose patients without ST elevation myocardial infarctionis no longer advised.^28^

Our meta-analysis found that post-arrest interventions such as ECMO after ROSC, pulmonary artery catheterisation monitoring, and emergent coronary angiography are associated with better neurological outcomes. However, this remained hypothesis-generating and may be influenced by confounding factors such as patient selection, institutional protocols, and care pathways. Modern post-cardiac arrest care emphasises active prevention of fever, with targeting a temperature of 37.5°C found to be effective as lower targets for comatose patients. The lack of clear association between targeted temperature management (TTM) and improved neurologic outcomes in our review may stem from high heterogeneity in patient demographics, variability in treatment protocols, and evolution of practice across different eras.

Our systematic review highlights risk stratification and protocol development for post-arrest care and should be interpreted cautiously due to low certainty of evidence and substantial heterogeneity. The shift towards prioritising neurological outcomes, rather than survival alone, marks a meaningful progression in cardiac arrest research. Structured post-cardiac arrest care should integrate rehabilitation, cognitive and emotional screening and comprehensive support for survivors. These advances highlight the importance of continuously updating clinical practices to align with emerging evidence and improve long-term outcomes for IHCA patients.

## 5 Strength and limitations

Key strengths include a comprehensive literature review and more diverse prognostic factors, which provides a robust dataset for analysis. The study followed a rigorous method for data collection, screening, and analysis, including the use of tools like QUIPS for bias assessment and GRADE approach for evaluating the certainty of our findings. We strictly adhere to recommendations for meta-analysis of prognostic studies and evaluate factors before arrest, during arrest, and after cardiac arrest. Given the observational design of most included studies, all reported relationships should be interpreted with caution. The exclusion of non-survivors introduces selection bias and the certainty of evidence is low. We reported several limitations such as the timing and standardisation of neurological assessment by three of the scoring methods were not the same for all studies. Although this introduces indirectness and heterogeneity, by utilising diverse timepoints and extended follow-up assessments post-ROSC enables a more precise understanding of the true neurological recovery timeline for survivors of IHCA. We have transparently documented the specific metrics and assessment intervals utilised in each study. However, the findings limit the comparability of the studies and do not address the quality of life or long-term functional outcomes of survivors. Secondly, the exclusion of non-survivors of cardiac arrest may lead to selection bias and overestimation of the effects of interventions and limit the generalisability of results to broader cardiac arrest population. Thirdly, non-standardised and non-structured data complicate extraction and synthesis potentially affecting the reliability of pooled analyses. The presence of low heterogeneity between small studies allowed for both random effects and fixed effects modelling along with heterogeneity assessments. While this heterogeneity limits the precision of pooled effect sizes, it also enhances the external validity of our findings, reflecting real-world clinical diversity. Despite missing data and mixed cohorts of OHCA and IHCA, sufficient information was available for meaningful analysis. Fourthly, most studies were observational, introducing potential unmeasured confounders and bias. The consistency in quality and methodology across studies supports the inferential strength of combined results. We recommend that future studies should adopt standardised neurological outcome measures and assessment intervals to improve methodological comparability and reliability. Inclusion of both survivors and non-survivors is essential to reduce selection bias and enhance generalisability. Addressing quality of life and long-term outcomes will provide a more comprehensive understanding of neurological recovery. The review includes studies up to 2022, excluding more recent clinical trials that could offer updated insights but maintains consistent methodology to ensure comparability and reduce selection bias. The adoption of COVID-19 protocols introduced systemic changes in cardiac arrest care, creating a new interim standard that complicates direct comparison between recent studies and pre-pandemic data. These pandemic-related modifications could influence the interpretation of recent evidence, highlighting the challenge of integrating newer findings into existing frameworks.^27^

## 6 Conclusion

This systematic review demonstrates that early recognition and management of reversible cardiac causes are associated with improved neurological outcomes in IHCA. Rigorous high-quality trials are needed to determine the causal impact and clinical significance of peri-arrest factors and interventions on the neurological recovery of IHCA.

## Data availability statement

This meta-analysis was conducted using summary data from previously published studies identified via systematic searches, with no new participant data collected. Full search strategies are included in the Supplementary material and data are available from the corresponding author upon request.

## Funding

This research did not receive any specific grant from funding agencies in the public, commercial, or not-for-profit sectors.

## Conflict of interest

We formally declare that this manuscript is original and has not been published before and is not currently being considered for publication elsewhere. There is no known conflict of interest associated with this publication and there is no financial support for this work that could have influenced its outcome.
