# Supplementary material for: Factors associated with good neurological outcomes in survivors of in-hospital cardiac arrest: A systematic review and meta-analysis
Source: Crit Care Resusc. 2026 Jul 28;28(3):100202. doi: 10.1016/j.ccrj.2026.100202 (PMC13449651; doi:10.1016/j.ccrj.2026.100202)
Supplement: Supplementary file 1 [file mmc1.docx]

**Supplementary Material**

**Figure S1.** Flowchart of the study selection process

**Figure S2** Factors of IHCA associated with good neurological outcomes

**Figure S3.** Pre-arrest Factors

Figure S3 (a): Age

Figure S3(b): Age comparison

Figure S3(c): Gender

Figure S3(d): Acute heart failure

Figure S3(e): Chronic ischaemic heart disease

**Figure S4.** Intra-arrest Factors

Figure S4 (a): PEA arrest

Figure S4 (b) Asystole

**Figure S5.** Post-arrest Factors

Figure S5 (a): Emergency coronary catheterization

Figure S5(b): Use of PA catheter

Figure S5 (c): ECMO after return of spontaneous circulation (ROSC)

**Table S1.** Studies selected for inclusion (n=15)

**Table S2.** Characteristics of study included in primary analysis

**Table S3.** Details of the included studies and outcomes

**Table S4.** Factors of IHCA not associated with neurologic outcome

**Table S5.** GRADE certainty of prognostic estimates

**Table S6.** GRADE level of certainty of the selected studies

**Table S7.** Quality Appraisal of the selected studies for the review

**Table S8.** Subgroup of Favourable IHCA factors and comparison between good and poor neurologic outcome

**Table S9.** Subgroup of Unfavourable IHCA factors and comparison between good and poor neurologic outcome

**Table S10.** Search strategy for various search engines

**PRISMA checklist**

**Funnel Plots**

**Figure S1.** Flowchart of the Study Selection Process

**Figure S2** Factors of IHCA associated with good neurological outcomes

**Figure** S**2**. Forrest plots showing pre-arrest factors, intra-arrest factors and post-arrest factors associated odds of good neurological outcome. Factors shown are male gender, telemetry monitoring, cardiac cause, acute myocardial infarction, chronic ischaemic heart disease, acute heart failure, initial rhythm VF/ VT arrest, emergent coronary catheterization, use of pulmonary artery catheter monitoring and ECMO after ROSC

**Figure S3. PREARREST FACTORS**

Figure S3 (a): Age

Random Effects Model: OR=0.93, 95% CI = 0.83 to 1.05, z=3.77, p=0.249

Heterogeneity: Х2=236.05, p=0.001, I2=99.86%, τ2=0.04

Egger: Bias=–0.86, p=0.579

Beggs: z=0.49, p=0.621

Figure S3(b): Age comparison

Random Effects Model: SMD=–0.27, 95% CI = –0.54 to 0.01, z=1.95, p=0.051

Heterogeneity: Х2=180.17, p=0.001, I2=92.20%, τ2=0.26

Egger: Bias=2.68, p=0.477

Beggs: z=1.29, p=0.198

Figure S3(c): Gender

Figure S3(d): Acute heart failure

Figure S3(e): Chronic ischaemic heart disease

**Figure S4. INTRA-ARREST FACTORS**

Figure S4 (a): PEA arrest

Figure S4 (b) Asystole

**Figure S5. POST ARREST FACTORS**

Figure S5 (a): Emergency coronary catheterization

Figure S5(b): Right heart catheterization or use of PA catheter

Figure S5 (c): ECMO after return of spontaneous circulation

**Table S1.** Studies selected for inclusion (n=15)

| **No.** | **Author/**  **Study Year** | **Study Design** | **Inclusion Period** | **Inclusion Criteria** |
| --- | --- | --- | --- | --- |
| 1 | Tujjar (14) | Retrospective | 01/2008  to 10/2012 | All comatose patients (Glasgow coma scale (GCS) score <9) admitted after in-hospital CA (IHCA) or out-of-hospital CA (OHCA) (January 2008 to October 2012) were included in an institutional database, if they survived for at least 48 h after CA, so that at least three serum creatinine (sCr) measurements were obtained |
| 2 | Wang (19) | Retrospective  (Glycaemic control) | 2006  to 2014 | Inclusion criteria: (1) age 18 years or older, (2) documented absence of pulse w/ performance of CPR for at least 2 min, (3) no documentation of a do-not-resuscitate order, and (4) achievement of sustained ROSC (i.e., ROSC ≥ 20 min without resumption of CPR |
| 3 | Wang (18) | Retrospective  (Haemoglobin level) | 2006  to 2012 | Inclusion criteria: (1) age 18 years or older, (2) documented absence of pulse with performance  of CPR for at least 2 min,  (3) No  documentation of a do-not-resuscitate order, and (4) achievement of sustained ROSC (i.e., ROSC ≥ 20 min without resumption  of chest compression. |
| 4 | Wang (17) | Retrospective  (DM cohort) | 2006  to 2015 | Inclusion criteria: (1) age 18 years or older  (2) documented absence of pulse with performance of chest compressions for at least 2 min, (3) no documentation of a do-not- resuscitate order, (4) presence of DM, and (5) a sustained ROSC (i.e., ROSC ≥20 min without resumption of chest compressions). |
| 5 | Wang (16) | Retrospective  (BMI, Thoracic AP diameter) | 2006  to 2015 | Inclusion criteria: (1) age ≥18 years, (2) documented absence of pulse with performance of CCs for at least 2 min, and (3) no documentation of a do- not-resuscitate order before arrest. |
| 6 | Storm (20) | Retrospective | 2006  to 2013 | All adult patients >18 years of age after cardiac arrest and assess for AKI occurrence after surviving cardiac arrest, admission to the ICU and receiving TTM. According to the KDIGO guidelines used in analysis with time frame consists of 48 h to 7 days. |
| 7 | Pound (21) | Prospective | 01/07/2017 to 09/08/2018 | All adults >=18 yo patients admitted as an acute care hospitalin-patient and experienced IHCA defined by period unresponsiveness with no observed respiratory effort and commencement of external compressions |
| 8 | Tran (22) | Prospective | 02/2012  to 01/2017 | All adult >=18 yo IHCA from all aetiologies Survived initial resuscitation Cerebral oximetry monitoring during the first 48 hour after IHCA in the ICU |
| 9 | Loza (4) | Prospective | 01/2014  to 06/2015 | All consecutive patients aged 18 years or older who had recovered from a first cardiac arrest either OHCA or IHCA after advanced life support measures admitted to the participating ICUs |
| 10 | Wang (15) | Retrospective  (Central obesity) | 2006  to 2015 | (1) age >=18-year (2) documented absence of pulse with performance of CPR for at least 2 min, (3) no documentation of DNR (4) achievement of sustained ROSC (i.e., ROSC ≥ 20 min without resumption of CPR |
| 11 | Chelly (23) | Retrospective | 01/2008  to 12/2014 | All unconscious  adult patients,  18 yo, successfully  resuscitated after an IHCA and admitted in ten French ICUs from January 2008 - December 2014 |
| 12 | Patel (24) | Prospective | 03/2014  to 05/2019 | All adult patients age >18 years old  With IHCA defined as cessation of heartbeat and respirations requiring  A resuscitation attempt lasting  > 5 minutes) |
| 13 | Andersson (2) | Retrospective | 2014  to 2018 | All adult >18 yo Skane region, Sweden (SWECRIT a regional biobank) |
| 14 | Halenarova (9) | Retrospective | 01/2012  to 12/2016 | Patients treated with ECPR for refractory IHCA or OHCA in the five centres were identified from medical charts and institutional ECMO databases from January 2012 to December 2016 |
| 15 | Jung (6) | Retrospective | 09/2016  to 08/2021 | All adults > =18 yo who had IHCA and achieved ROSC for at least 20 consecutive mins |

**Table S2.** Characteristics of study included in primary analysis

**Table S3.** Details of the included studies and outcomes

| **Study No. /Author/ Year** | **Number of Eligible Participants** | **Follow up duration included in analysis** | **Outcome Measure (CPC category mRS scale Glasgow Outcome score)** | **Outcome No. of Favourable CPC 1-2 mRS <=3 GOS 4-5** | **Outcome No. of Unfavourable**  **CPC 3-5 mRS scale>=4, GOS 1-3** | **CPR Duration (mins) Mean SD (Favourable NO)** | **CPR**  **Duration**  **(mins)**  **= /Mean SD**  **(Unfavourable**  **NO)** |
| --- | --- | --- | --- | --- | --- | --- | --- |
| 1.Tujjar 2015 | 199 | 3 months | Primary (CPC) | 81  (40.7%) | 118  (59.3%) | 13 (7-21)  81 pts | 18 (10-27)  118 pts |
| 2.Wang 2016  (Glycaemic control) | 402 | hospital discharge | Primary (CPC) | 70  (17.4%) | 332 (82.6%) | 12.7 (10.2)  70 pts | 19.3 (16.7)  332 pts |
| 3.Wang 2016  (Haemoglobin level) | 426 | hospital discharge | Primary (CPC) | 54  (12.7%) | 372 (87.3%) | 9 (5 -14.8)  54 pts | 14 (7-26)  372 pts |
| 4.Wang 2018  (Diabetic cohort) | 141 | hospital discharge | Primary (CPC) | 30  (21.3%) | 111 (78.7%) | 12.1 (9.2)  30 pts | 24.6 (43.4)  111 pts |
| 5.Wang 2018  (BMI, Thoracic AP Diameter) | 766 | hospital discharge | Primary (CPC) | 58  (7.57%) | 708 (92.4%) | 12.4 (10.8)  58 pts | 32.2 (29.8)  708 pts |
| 6.Storm 2018 | 503 | hospital discharge | Primary (CPC) | 246  (48.9%) | 257  (51%) | 15 (10-22)  246 pts | 20 (12-28)  257 pts |
| 7.Pound 2020 | 152 | hospital discharge | Primary (mRS) | 43  (28.3%) | 109  (71.7%) | 1 (0.5-4)  43 pts | 11(5-20)  109 pts |
| 8.Tran 2020 | 87 | hospital discharge | Primary  (CPC) | 26  (29.9%) | 61  (70.1%) | 12 +/-11.5  26 pts | 10 +/- 11  61 pts |
| 9.Loza 2020 | 243 | hospital discharge | Primary (CPC) | 221  (91%) | 22  (9%) | 14 (6-25)  221 pts | 25.5(15-38)  22 pts |
| 10.Wang 2020  (Central Obesity) | 648 | hospital discharge | Primary (CPC) | 47 (7.25%) | 601 (92.75%) | 13.3 (10.9)  47 pts | 32.5 (29)  601 pts |
| 11.Chelly 2020 | 381 | hospital discharge | Primary (CPC) | 125  (32.8%) | 256  (67.2%) | 11 (9-13)  125 pts | 18 (16-21)  256 pts |
| 12.Patel 2021 | 105 | hospital discharge | Primary (GOS) | 27  (25.7%) | 78  (74.3%) | 10.2 (8.9)  27 pts | 19.9(8.8)  78 pts |
| 13.Andersson  2022 | 245 | 2 to 6 months after cardiac arrest | Primary (CPC) | 98  (40%) | 146  (60%) | 7 (5-15)  98 pts | 11(6-20)  146 pts |
| 14.Halenarova  2022 | 163 | 3 months | Primary  (CPC) | 56  (34.4%) | 107 (65.6%) | 34 (20-33)  Time from arrest to ECMO (min)  56 pts | 50 (36-64)  Time from arrest to ECMO (min)  107 pts |
| 15. Jung 2023 | 488 | hospital discharge | Primary (CPC) | 85  (17.4%) | 403 (82.6%) | 5.0 (2.5-9)  85 pts | 10.0 (5-17)  403 pts |

| **Table S4.** Factors of IHCA not associated with neurologic outcome | | | | | | |
| --- | --- | --- | --- | --- | --- | --- |
|  | **Studies** | **Patients (n)** | **Pooled OR, SMD*** | **95% CI** | **P value** | **I2**  **(%)** |
| Witnessed arrest | 10 | 3339 | 1.41 | 0.81, 2.43 | 0.22 | 76% |
| Arrest at night | 5 | 2383 | 0.88 | 0.66, 1.12 | 0.35 | 0 |
| Arrest on weekend | 5 | 2383 | 0.93 | 0.70, 1.25 | 0.65 | 0 |
| Arrest in ICU | 8 | 3233 | 1.03 | 0.74, 1.43 | 0.87 | 49% |
| Hypertension | 3 | 824 | 0.90 | 0.68, 1.2 | 0.49 | 0 |
| T2 Diabetes mellitus | 9 | 3717 | 1.10 | 0.91, 1.34 | 0.33 | 0 |
| COPD | 2 | 892 | 0.85 | 0.46, 1.54 | 0.58 | 0 |
| Chronic heart failure | 6 | 2582 | 1.29 | 0.88, 1.9 | 0.19 | 46% |
| End-stage renal failure (on dialysis) | 7 | 2700 | 0.96 | 0.69, 1.34 | 0.82 | 0 |
| Stroke | 6 | 2871 | 0.66 | 0.36, 1.20 | 0.17 | 0 |
| Renal failure during admission | 3 | 449 | 0.87 | 0.46, 1.66 | 0.67 | 56% |
| Use of mechanical ventilation | 6 | 2644 | 0.81 | 0.60, 1.10 | 0.17 | 0 |
| Temperature management | 9 | 3199 | 1.02 | 0.53, 1.96 | 0.95 | 61% |
| Use of Vasopressors | 7 | 2807 | 0.80 | 0.64, 1.01 | 0.05 | 0 |
| Anti-arrhythmic drugs use | 4 | 1957 | 1.12 | 0.53, 2.37 | 0.77 | 55% |
| Unspecified ward | 8 | 3496 | 0.66 | 0.44, 0.98 | 0.42 | 76% |
| Post-arrest Glucose level | 3 | 630 | -0.09* | -0.40,0.23 | 0.58 | 55% |
| Post-arrest PaO2 level | 2 | 513 | 0.08* | 0.16, 0.33 | 0.49 | 0 |

**Table S5.** GRADE Certainty of Prognostic Estimates

(Prearrest, Intra-arrest, Post arrest factors)

Adapted from Iorio A et al. Use of GRADE for assessment of evidence about prognosis: rating confidence in estimates of event rates in broad categories of patients. BMJ 2015; 350 (26)

**Table S6.** GRADE level of certainty of the selected studies n=15

| **Study** | **Study**  **Design** | **Risk of Bias** | **Consistency** | **Directness** | **Precision** | **Publication**  **bias** | **Level of**  **Certainty** |
| --- | --- | --- | --- | --- | --- | --- | --- |
| **Prospective Study** |  | | | | | |  |
| Pound | Moderate | Moderate | Moderate | Low | Low | Low | Moderate |
| Patel | Moderate | Moderate | Low | Low | Low | Low | Low |
| Loza | Low | Moderate | Low | Moderate | Low | Low | Low |
| Tran | Low | Moderate | Moderate | Moderate | Low | High | Low |
| **Retrospective** |  | | | | | |  |
| Jung | Moderate | Low | Moderate | Moderate | Moderate | Moderate | Moderate |
| Andersson | Low | Moderate | Moderate | Moderate | Moderate | Low | Moderate |
| Storm | Moderate | Moderate | Moderate | Moderate | Low | Low | Moderate |
| Wang  (Diabetic  group) | Low | Moderate | Moderate | Moderate | Low | Moderate | Low |
| Wang  (BMI, Thoracic AP subgroup) | Low | Moderate | Moderate | Moderate | Moderate | Moderate | Very Low |
| Wang (Blood glucose control) | Low | Moderate | Low | Moderate | Moderate | Low | Low |
| Wang (Central Obesity) | Moderate | Low | Moderate | Low | Moderate | Low | Low |
| Wang (Haemoglo  bin level) | Low | Moderate | Low | Moderate | Low | Low | Low |
| Tujjar | Moderate | Low | Low | Low | Moderate | Low | Low |
| Hallenarova | Low | Moderate | Low | Moderate | Low | Moderate | Low |
| Chelly | Moderate | Moderate | Low | Moderate | Moderate | Moderate | Moderate |

**Table S7.** Quality Appraisal of the selected studies for the review n =15

Hayden J, Cote P, Bombardier C. Evaluation of the quality of prognosis studies in systematic reviews. Ann Intern Med 2006; 144:427-431 (25)

| **Study** | **Study Participation** | **Study**  **Attrition** | **Prognostic**  **Factor Measurement** | **Outcome Measurement** | **Confounding Measurement Account** | **Analysis and Reporting** | **Overall Rating** |
| --- | --- | --- | --- | --- | --- | --- | --- |
| **Prospective Study** |  | | | | | |  |
| Pound | Low | Moderate | Moderate | Low | Low | Low | Low |
| Patel | Low | Moderate | Low | Moderate | Moderate | Low | Moderate |
| Loza | Low | Moderate | Low | Moderate | Moderate | Low | Moderate |
| Tran | Moderate | Moderate | Low | Low | Moderate | Low | Moderate |
| **Retrospective**  **Study** |  | | | | | |  |
| Jung | Moderate | Low | Low | Low | Moderate | Low | Low |
| Andersson | Low | Moderate | Moderate | Low | Moderate | Low | Moderate |
| Storm | Moderate | Low | Moderate | Low | Moderate | Low | Moderate |
| Wang  (Diabetes  cohort) | Low | Moderate | Low | Moderate | Moderate | Low | Moderate |
| Wang  (BMI, Thoracic AP subgroup) | Low | Low | Low | Moderate | Moderate | Low | Low |
| Wang (Blood glucose level) | Moderate | Moderate | Low | Low | Moderate | Low | Moderate |
| Wang (Central Obesity) | Moderate | Low | Moderate | Low | Moderate | Low | Moderate |
| Wang (Haemoglobin level) | Moderate | Low | Low | Moderate | Moderate | Low | Moderate |
| Tujjar | Moderate | Low | Low | Low | Moderate | Low | Moderate |
| Hallenarova | Moderate | Low | Moderate | Moderate | Low | Low | Moderate |
| Chelly | Moderate | Low | Low | Low | Moderate | Low | Low |

**Table S8**. Subgroup of Favourable IHCA factors and comparison between with good and poor neurologic outcome

| **Subgroup**  (Number  of studies) | **Good neurological outcome** | **Poor neurological outcome** | **OR**  (95% CI) | **P value** | **I^2^** |
| --- | --- | --- | --- | --- | --- |
| Cardiac cause (6) | 551/827 | 397/906 | 2.12  (1.54, 2.92) | 0.001 | 46% |
| Male Gender (15) | 896/1267 | 2273/3681 | 1.425 (1.136,1.788) | 0.002 | 46 % |
| Chronic Ischaemic Heart Disease (6) | 50/ 357 | 129/2270 | 1.84  (1.12, 3.02) | 0.016 | 38% |
| Acute MI (5) | 55/274 | 208/2195 | 2.14  (1.53, 2.99) | 0.001 | 0 |
| Acute Heart Failure (4) | 61/189 | 324/1792 | 1.99  (1.42, 2.78) | 0.001 | 0 |
| Arrest in Telemetry (10) | 402/622 | 1696/3007 | 1.34 (1.00,1.78) | 0.048 | 44% |
| Shockable Rhythm (VF/VT) (13) | 422/965 | 502/3317 | 3.00 (2.30,3.91) | 0.001 | 48% |
| ECMO after ROSC (7) | 105/491 | 292/2494 | 1.71  (1.20, 2.43) | 0.003 | 0 |
| Pulmonary artery catheter (4) | 8/205 | 17/1752 | 3.84  (1.51, 9.75) | 0.005 | 0 |
| Emergency Coronary Catheterisation (7) | 151/506 | 100/2207 | 4.55  (2.11, 9.82) | 0.001 | 80% |

**Table S9.** Subgroup of Unfavourable IHCA factors and comparison between good and poor neurologic outcome

| **Subgroup**  (Number  of studies) | **Good neurological outcome** | **Poor neurological outcome** | **OR** (95% CI) or **SMD**† | **P value** | **I^2^** |
| --- | --- | --- | --- | --- | --- |
| Age (14) | 558/3078 | 2520/3078 | SMD -0.18†  (-0.28, -0.08 | 0.001 | 0 |
| Duration of CPR mins (14) | 232/2062 | 1830/2062 | SMD -0.55†  (-0.69, -0.41) | 0.001 | 1% |
| PEA (6) | 195/404 | 661/1053 | OR 0.45 (0.25,0.81) | 0.008 | 78% |
| Asystole (4) | 55/252 | 213/719 | OR 0.63  (0.42, 0.96) | 0.033 | 24% |
| Malignancy (7) | 39/442 | 774/2673 | OR 0.23  (0.10, 0.52) | 0.001 | 73% |
| Liver disease (8) | 58/481 | 574/2752 | OR 0.62  (0.44, 0.88) | 0.007 | 12% |
| Respiratory insufficiency (8) | 210/481 | 1915/2752 | OR 0.40 (0.21,0.75) | 0.004 | 84% |
| Sepsis (6) | 30/344 | 354/2527 | OR 0.49  (0.32, 0.74) | 0.001 | 0 |
| Lower haemoglobin post-arrest (2) | 80/513 | 433/513 | SMD 0.37† (0.03, 0.71) | 0.03 | 39% |
| High Charlson Comorbidity Index (3) | 135/1555 | 1420/1555 | SMD -0.57†  (-0.75, -0.39) | 0.001 | 0 |

**Table S10.** Search strategy for various search engines

**Embase:** “heart arrest” OR “cardiopulmonary arrest” OR “hospitalisation” OR in-hospital” OR IHCA AND “Survivor” OR “clinical trial” OR “6 month” OR “12 month”

**Medline:** “heart OR cardiac OR “cardiopulmonary arrest” OR IHCA AND “Resuscitation”, “Inpatient” OR IHCA AND/ OR “Survivor” OR “controlled” OR “multicentre” AND/OR “6 month”/ “12 month”

**Google scholar:** cardiac/heart/ cardiopulmonary arrest/ “in-hospital”/inpatient/ hospitalised/survival/ “6 month”/ “12 month”

 Key Components of a Standardised Search Strategy

- **Research Question Definition (PICO):** The search is structured around Population/Problem, Intervention, Comparison, and Outcome (PICO) to identify key concepts.
- **Controlled Vocabulary (Subject Headings):** Use of indexed, hierarchical terms specific to databases (e.g., Mesh in PubMed, Emtree in Embase) to ensure all relevant articles are found, regardless of the keywords used by authors.
- **Keywords and Free-Text Terms:** Using synonyms, variations in spelling (e.g., UK/US), abbreviations, and truncation (e.g., medic* for medicine, medical) in title/abstract fields.
- **Boolean Operators and Nesting:** Combining keywords and subject headings using AND (to narrow) and OR (to broaden), using parentheses to group synonyms.
- **Field Tags:** Limiting searches to specific fields like Title, Abstract, or Author to improve precision (e.g., [tiab] in PubMed)

___________________________________________________________________________Limits applied in Medline, English language, humans, publication year 2020 to 2022, prognosis best balance sensitivity, specificity. Age group, all adults 19 plus years, for Google scholar, English language, year limit, words “heart arrest’ appear in the article. Unfortunately google scholar is less systematic in search terminologies.

PRISMA Checklist

**Funnel Plots**

**i. Factors associated with good neurologic outcome**

Cardiac Cause

Gender and Good CPC

*Fixed Effects Model: OR=1.35, 95% CI = 1.15 to 1.57, z=3.77, p=0.001*

*Heterogeneity: Х2=26.95, p=0.020, I2=48.00%*

*Egger: Bias=1.26, p=0.022*

*Beggs: z=2.28, p=0.023*

CHF during admission

AMI during admission

Chronic Ischaemic Heart disease (Prior MI)

Coronary Angiogram Performed

Use of PA Catheter

ECMO after ROSC

Initial shockable rhythm (VF/VT)

Arrest in Telemetry

**ii. Factors associated with poor neurologic outcome**

Age

Random Effects Model: OR=0.93, 95% CI = 0.83 to 1.05, z=3.77, p=0.249

Heterogeneity: Х2=236.05, p=0.001, I2=99.86%, τ2=0.04

Egger: Bias=–0.86, p=0.579

Beggs: z=49, p=0.621

Duration of CPR

Random Effects Model: OR=0.80, 95% CI = 0.67 to 0.97, z=–2.32, p=0.020

Heterogeneity: Х2=716.28, p=0.001, I2=99.88%, τ2=0.11

Egger: Bias=–4.72, p=0.137

Beggs: z=1.75, p=0.08

Asystole arrest

PEA arrest

Sepsis

Respiratory Insufficiency

Hepatic Insufficiency

Malignancy

Low Haemoglobin level

High Charlson Comorbidity Index

**iii. Factors not associated with neurologic outcome**

Anti-arrhythmic drugs

Arrest at night

Arrest at weekend

COPD

Development of Renal failure

Diabetes

Dialysis

Hypertension

Arrest in ICU

Mechanical ventilation

Post-arrest Glucose

Post-arrest pO2 level

Previous Stroke

Prior Heart failure

Targeted Temperature Management

Vasopressor use

Witnessed arrest

Unspecified ward

IABP
